# Targeting PARK7 Improves Acetaminophen-Induced Acute Liver Injury by Orchestrating Mitochondrial Quality Control and Metabolic Reprogramming

**DOI:** 10.3390/antiox11112128

**Published:** 2022-10-28

**Authors:** Jiao Cai, Deqin Kong, Zi Long, Jiangzheng Liu, Rui Liu, Chunxu Hai

**Affiliations:** Department of Military Toxicology and Chemical Defense Medicine, Shaanxi Provincial Key Lab of Free Radical Biology and Medicine, The Ministry of Education Key Lab of Hazard Assessment and Control in Special Operational Environment, School of Public Health, Air Force Medical University, Xi’an 710032, China

**Keywords:** PARK7, mitochondrial quality control, APAP, acute liver injury

## Abstract

Mitochondrial dysfunction and oxidative stress are considered to be key events in acetaminophen (APAP)-induced acute liver injury. Mitochondrial quality control, including mitophagy and mitochondrial synthesis, can restore mitochondrial homeostasis and thus protect the liver. The role of PARK7, a mitochondrial stress protein, in regulating mitochondrial quality control in APAP-induced hepatotoxicity is unclear. In this study, L02 cells, AML12 cells and C57/BL6 mice were each used to establish models of APAP-induced acute liver injury. PARK7 was silenced in vitro by lentiviral transfection and knocked down in vivo by AAV adeno-associated virus. Changes in cell viability, apoptosis, reactive oxygen species (ROS) level, serum enzyme activity and pathological features were evaluated after APAP treatment. Western blotting, real-time PCR, immunofluorescence, electron microscopy and Seahorse assays were used to detect changes in key indicators of mitochondrial quality control. The results showed that APAP treatment decreased cell viability and increased the apoptosis rate, ROS levels, serum enzyme activity, pathological damage and PARK7 expression. PARK7 silencing or knockdown ameliorated APAP-induced damage to the cells and liver. Furthermore, PARK7 silencing enhanced mitophagy, increased mitochondrial synthesis, and led to a switch from oxidative phosphorylation to glycolysis. Taken together, these results suggest that PARK7 is involved in APAP-induced acute liver injury by regulating mitochondrial quality control and metabolic reprogramming. Therefore, PARK7 may be a promising therapeutic target for APAP-induced liver injury.

## 1. Introduction

Drug-induced liver injury has become a public health problem that cannot be ignored with the increasing number of reports of liver injury caused by various drugs. Acetaminophen (APAP) is a widely used antipyretic and analgesic drug and is the drug most closely related to liver damage. In view of the worldwide spread of COVID-19, many patients in clinical practice have been taking APAP-containing drugs to relieve headache and fever [1], increasing the risk of APAP poisoning. Currently, N-acetyl cysteine (NAC) is a clear and effective antidote for APAP poisoning, but it can only be used in the early stage of the disease and thus has a narrow treatment window [2]. In the United States, APAP has become the most commonly used drug that causes acute liver failure [3], and hepatotoxicity caused by APAP has also been reported in Asian populations [4]. In China, APAP is an important factor second only to hepatitis B virus in causing liver failure. The mechanism of hepatotoxicity of APAP has not been fully elucidated, but oxidative stress and mitochondrial dysfunction are recognized as important mechanisms of APAP-induced liver injury [5,6]. Mitochondria are key targets of APAP-induced hepatotoxicity [7]. The intermediate NAPQI formed by excessive APAP in metabolism covalently binds with mitochondrial proteins, leading to mitochondrial oxidative stress and dysfunction and ultimately to liver cell death [8,9].

As important organelles of eukaryotic cells, mitochondria are subject to strict quality control [10]. Mitochondrial quality control eliminates mitochondrial damage or improves mitochondrial quality control mechanisms and can restore mitochondrial homeostasis and benefit liver health [11]. For example, it can improve liver fibrosis by regulating mitophagy and mitochondrial biogenesis [12]. Autophagy is an intracellular pathway that maintains intracellular homeostasis by lysosomal degradation of intracellular macromolecules and damaged or excess organelles. Mitochondrial autophagy, a form of autophagy, is an evolutionarily conserved mechanism used for mitochondrial quality control and homeostasis [13]. Autophagy is a key quality control mechanism that maintains liver homeostasis in both parenchymal and nonparenchymal liver cells [14]. Autophagy disorders have been widely studied in liver diseases, such as alcoholic liver injury, hepatocellular carcinoma and viral hepatitis [15]. It has been reported that removal of APAP protein adducts through autophagy can protect against APAP-induced liver injury in mice [16]. Pharmacological inhibition of autophagy may aggravate APAP-induced liver injury [17]. Mitochondria are the energy source of cells, and APAP-induced mitochondrial autophagy is an important mechanism to maintain mitochondrial homeostasis, which can reduce ROS production and decrease the oxidative stress level of liver cells by removing damaged mitochondria [18]. However, the regulatory mechanism of mitochondrial autophagy in the context of APAP hepatotoxicity remains unclear.

APAP induces changes in mitochondrial stress proteins following mitochondrial stress, including PRDX6, VDAC2, HSP70 and PARK7. The change in PARK7 is the most obvious. PARK7 was identified as a mitochondrial stress protein in the primary 3D human liver microtissue proteome after APAP exposure [19]. PARK7 is a protein-coding gene that has been studied in the context of early Parkinson’s disease [20]. The PARK7 protein is involved in a variety of biological pathways, including oxidative stress [21] and mitochondrial regulation [22]. In CCL4-induced liver fibrosis, PARK7 deficiency alleviates fibrosis in mice by inhibiting ROS production and liver damage [23]. However, how PARK7 regulates mitochondrial quality control in APAP-induced acute liver injury has not been studied.

At present, the incidence of APAP liver injury is high, and the mechanism is unclear, but it is closely related to mitochondria and ROS, which can cause mitochondrial stress and changes in mitochondrial quality control, and mitochondrial stress-related molecules, including PARK7. In this study, we aimed to observe changes in mitochondrial quality control regulated by PARK7 and explore its role in the hepatotoxicity of APAP. We found that excessive APAP can increase the expression of PARK7, while silencing PARK7 can protect against liver injury. Mechanistically, PARK7 protects against liver injury by promoting autophagy and mitochondrial synthesis. Therefore, our results suggest that PARK7 may be a potential therapeutic target for APAP-induced acute liver injury.

## 2. Materials and Methods

### 2.1. Materials

APAP (Bioxtra, ≥99.0%) was purchased from Sigma-Aldrich (St. Louis, MO, USA). RPMI-1640 medium, dual antibodies (penicillin and streptomycin), PBS, and 0.25% trypsin were purchased from HyClone. Fetal bovine serum was purchased from Gibco. CCK-8 was purchased from Nanjing Enjing Biotechnology. An Annexin V/PI double staining apoptosis kit and nuclear protein extraction kit were purchased from Nanjing Kaiji Biotechnology. Autophagy double standard adenovirus reagent was purchased from Han Heng Biotechnology. A BCA protein quantitative kit, JC-1 and MitoSox were purchased from Thermo Fisher. MitoTracker Red and transfection reagent were purchased from Invitrogen. The qPCR kit was purchased from AG Biotechnology, and the small interfering RNA product was purchased from Beijing Qing Ke Biotechnology. The primary antibodies used in this study included PARK7 (Abcam, #ab76008), LC3B (Abcam, #ab192890), P62 (Abcam, #ab109012), Keap1 (Abcam, #ab227828), Nrf2 (Abcam, #ab62352), PGC1-α (Affinity, #AF5395), NRF1 (Affinity, #AF5298), TFAM (Affinity, #AF0531), β-tubulin, actin, and TBP antibodies and goat anti-rabbit IgG and goat anti-mouse IgG antibodies, which were purchased from Affinity. All other chemicals were analytical grade chemicals provided by commercial suppliers.

### 2.2. Cell Lines and Experimental Animals

The human normal liver cell line L02 was provided by Shanghai Institute of Cell Biology, Chinese Academy of Sciences, using RPMI-1640 medium containing 10% FBS and 1% double antibody. The mouse normal liver cell line AML12 was purchased from Wuhan Punosai Life Science and Technology. The medium composition was DMEM/F12 + 40 ng/mL dexamethasone + insulin–transferrin–selenium + 10% FBS + 1% P/S Solution. Both kinds of cells were cultured at 37 °C, CO_2_ volume fraction 5% and saturated humidity. The cells in logarithmic growth phase were digested with 0.25% trypsin and then neutralized in medium with an equal volume. The cells were beaten evenly and diluted into a single cell suspension for use.

Male SPF C57/BL6 mice (weight 19–22 g, Certification No. SCXK(Shaanxi)2019-001) were purchased from the Experimental Animal Center of Air Force Military Medical University. Mice were randomly divided into four groups (10 in each group) with 0 mg/kg, 300 mg/kg, 400 mg/kg and 500 mg/kg APAP and treated with APAP or normal saline. Mice in the control group were injected with normal saline and sacrificed 24 h later. The mice were deprived of water and fasted the night before administration.

### 2.3. Mice Infected with AAV Adeno-Associated Virus

The livers of mice were infected with adeno-associated virus type 9 (Han Heng Biotechnology, Shanghai, China). Male SPF C57/BL6 mice aged 4–5 weeks were placed in a fixator with their tails exposed. The tails of the mice were wiped with an alcohol-soaked cotton ball to dilate their blood vessels. The virus was thawed on ice, and 100 µL of virus was injected into the tail vein with a microsyringe. The infection efficiency was detected after 3 weeks of infection.

### 2.4. Serum Analysis and Histological Examination

The blood was placed at room temperature for 2 h and centrifuged at 3000× *g* for 20 min. Serum was collected, and ALT and AST levels were determined by a kit. Mice were anaesthetized by isoflurane inhalation, and liver tissue was removed. Part of the mice were fixed with 4% paraformaldehyde, dehydrated, embedded, sliced (LeicaRM2135 rotary slicer, 4 μm) and stained with haematoxylin-eosin(HE). In addition, paraffin sections were used for immunohistochemical staining, routinely dewaxed, incubated with 3% H_2_O_2_ for 20 min, washed with distilled water, sealed with serum, incubated with primary antibody and secondary antibody, stained with DAB chromogenic agent, rinsed with water and sealed with tablets. Finally, changes in the liver tissues of mice were observed under a positive optical microscope (Nikon, Tokyo, Japan).

### 2.5. Frozen Section ROS and TUNNEL Experiments

Frozen sections were rewarmed at room temperature, and moisture was controlled. A circle was drawn around the tissue, and autofluorescence quencher was added to the circle for 5 min. The circle was then rinsed with running water for 10 min. ROS staining solution was added to the circle, and the cells were incubated at 37 °C for 30 min in the dark. Then, the nuclei were counterstained with DAPI, and the slices were sealed. Finally, the slices were observed under a fluorescence microscope, and images were collected.

Frozen sections were fixed in 4% paraformaldehyde for 30 min, washed 3 times with PBS, incubated with proteinase K working solution at 37 °C for 22 min, washed 3 times with PBS, incubated with membrane breaking working solution, for 20 min, washed 3 times with PBS, and then processed according to the instructions of the TUNNEL kit. The images were observed and collected under a fluorescence microscope.

### 2.6. Detection of the Cell Damage Index

Single-cell suspensions were inoculated in 96-well plates, 6-well plates and laser confocal dishes at the appropriate density, and the cells were mixed evenly during inoculation. After all the cells adhered to the wall, different groups were treated with drugs for 24 h, and 96-well plates were treated with CCK-8 working solution at 37 °C for 2 h. The absorbance at 450 nm was determined by an all-band microplate analyzer (Infinite M200 PRO, Austria). The survival rate of L02 cells in each group was calculated according to the instructions of the CCK-8 kit. Cells in the six-well plates were washed twice with PBS, and 500 μL binding buffer, 5 μL of Annexin V and 5 μL of PI were added to each sample according to the instructions of the Annexin V/PI kit. The cells were incubated at room temperature in the dark for 15 min. Flow cytometry (BD Accuri C6, Piscataway, NJ, USA) was used to detect apoptosis. Software analysis was used to draw a two-dispersion dot plot, with FITC as the abscissa and PI as the ordinate. The apoptosis rate was calculated as the sum of the percentages of early and late apoptosis. The laser confocal dishes were stained according to the operating instructions of the MitoSox kit and JC-1 kit (mitochondrial ROS and mitochondrial membrane potential were detected, respectively) and incubated at 37 °C for 30 min in the dark. The staining solution was discarded, the cells were washed with PBS twice, and laser confocal imaging (Olympus, Shinjuku City, Japan) was used.

### 2.7. Western Blotting and qPCR

Total protein was extracted with RIPA lysis buffer, and the liver tissue was thoroughly ground in a tissue grinder (Xinzhi Bio, Ningbo), followed by ice lysis for 30 min and centrifugation at 12,000× *g* for 20 min. The supernatant was preserved. Nuclear protein was extracted with a nuclear protein extraction kit, washed twice with PBS, and then analyzed according to the kit instructions. Protein samples were separated and transferred to PVDF membranes by SDS—PAGE (Bio-Rad, Hercules, CA, USA) and blocked with skim milk for 2 h. Primary antibody was added overnight at 4 °C. Secondary antibodies were incubated the next day, washed with PBS, and developed by automatic chemiluminescence (Bio-Rad, Hercules, CA, USA).

Total RNA was extracted from cells or tissues using a formulated lysed Buffer reagent with a SteadyPure Generic RNA Extraction Kit (AG, AG21017). cDNA was synthesized using an Evo M-MLV reverse transcription kit (AG, AG11706). A LightCycler sequence detection system (BIO-RAD, CFX96, USA) was used for qRT—PCR analysis. The cycle parameters were as follows: 95 °C for 30 s, 95 °C for 5 s 40 times, and 60 °C for 30 s 40 times. Finally, CFX Manager 2.1 software (Bio-Rad, Hercules, CA, USA) was used for analysis.

### 2.8. Cell Transfection Experiment

The PARK7 gene accession numbers are NM _007262.5 (human) and NM _020569.3 (mouse). L02 cells were transfected with lentivirus. Prior to transfection, 6–8 × 10^6^ cells were seeded in a 10 cm cell culture dish and cultured in a 5% CO_2_ incubator at in 37 °C. After 16–24 h, the cell density reached 80–90%, and transfection was performed. The vector containing the shRNA of the target gene, the lentiviral packaging vector and buffer were mixed in Eppendorf tubes, and the mixture was evenly added to the 10 cm cell culture dish drop by drop and gently mixed. The cells were incubated in a 5% CO_2_ incubator at 37 °C. After 16 h of culture, the medium was discarded and replaced with 10 mL of fresh complete medium. At 24 h after fluid exchange, the supernatant was collected and stored in a refrigerator at 4 °C, 10 mL of fresh complete medium was added to the 10 cm cell culture dish, and cell culture was continued at 37 °C in a 5% CO_2_ incubator. The supernatant was collected again, mixed with the supernatant from the first collection, and centrifuged at 1000× *g* rpm for 5 min. The cell debris was discarded, and the supernatant was filtered through a 0.45 μm PVDF filter into a 50 mL round-bottom centrifuge tube. After high-speed centrifugation at 50,000× *g* for 2 h at 4 °C, the virus precipitate was resuspended by adding PBS at 200 μL/10 cm petri dish, packaged into clean 1.5 mL Eppendorf tubes at 100 μL per tube and stored in a refrigerator at −80 °C.

AML12 cells were transfected with siRNA products, and the target gene siRNA was prepared into 20 μmol/L storage solution with sterilized ddH_2_O and placed on ice. Then, AML12 cells were transfected with the Park7 gene for siPARK7 silencing. The primer sequence information is as follows: 

 M57320-siPark7-1-ss: GCACAGAAUUUAUCUGAGUTT, 

 M57320-siPark7-1-as: ACUCAGAUAAAUUCUGUGCTT. 

 M57320-siPark7-2-ss: GCUUGUUCUCAAAGACUAGTT, 

 M57320-siPark7-2-as: CUAGUCUUUGAGAACAAGCTT. 

 M57320-siPark7-3-ss: GUAAUGAUUUGUCCAGAUATT, 

 M57320-siPark7-3-as: UAUCUGGACAAAUCAUUACTT.

### 2.9. Immunofluorescence

The cells in the confocal petri dish were washed with PBS and fixed with 4% paraformaldehyde for 30 min. After washing with PBS three times, the membrane was lysed with 0.2% Triton X-100-PBS for 10 min and sealed with serum-containing medium for 1 hour. The cells were incubated with the primary antibody overnight. The next day, the fluorescence secondary antibody was added and incubated for 30 min, and the nuclei were stained with DAPI. After adding an appropriate amount of anti-fluorescence quenching agent, the images were collected by laser confocal scanning.

### 2.10. Electron Microscopy

The cell samples were washed with PBS 3 times, fixed with 3% glutaraldehyde for 2 h, washed with PBS 3 times, fixed with 1% OsO_4_ for 1 h, and then subjected to gradient dehydration and embedding treatment. Ultrathin sections were finally made and observed by electron microscopy.

A small piece of liver tissue was fixed, dehydrated and embedded by infiltration. The embedding plate was placed in a 60 °C oven for polymerization for 48 h, and the resin block was removed for use. Ultrathin sections were prepared and stained. The copper mesh sections were dried overnight at room temperature in the copper mesh box. Images were collected and analyzed under a transmission electron microscope (Hitachi, Tokyo, Japan).

### 2.11. Autophagy Flux Detection

The cells were inoculated into a confocal dish, and when the cells adhered to the wall and the density reached 40%, the autophagy double-label adenovirus (MRFP-GFP-LC3) was transfected with light isolation for 4 h. When the cell density reached 80%, the cells were treated with APAP. After 24 h, the solution was discarded, and the cells were fixed with 4% paraformaldehyde for 10 min, washed with PBS three times, and photographed under a laser confocal microscope.

### 2.12. Seahorse Experiment

Cells in each group (100 µL at a density of 10^5^ cells/mL) were seeded in 24-well plates with 150 mL of growth medium and incubated at 37 °C until they adhered. After that, 175 µL of the original growth medium was aspirated, and the cells were washed twice with 600 µL of special test medium. Finally, 450 µL to 525 µL of medium was added to observe the energy consumption of cells in each well under a microscope. After that, the cells were placed in a non-CO_2_ incubator and incubated for 1 hour. Then, 75 µL APAP was added to four replicate wells per condition according to the experimental design. Finally, the instrument method was edited, the calibration plate was used for calibration, and then the calibration plate was replaced with the cell plate to run the test (Agilent Technologies, Santa Clara, CA, USA).

### 2.13. Statistical Analysis

Experimental data were processed and analyzed using GraphPad Prism 8.0. One-way ANOVA was used for comparisons between groups, and the data are expressed as the mean ± SD. *p* < 0.05 was considered statistically significant.

## 3. Results

### 3.1. Establishment of the APAP Acute Liver Injury Model

**Figure 1:**
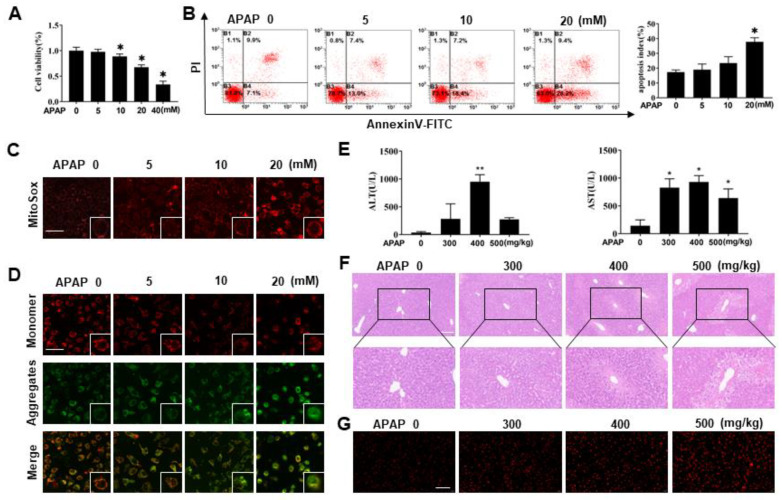
Establishment of the APAP acute liver injury model. (**A**) Changes in cell viability were measured after the L02 cells were treated with a gradient of concentrations of APAP, N = 6. * *p* < 0.05 vs. the Ctr group. (**B**) The apoptosis of L02 cells was detected by AnnexinV/PI double staining, N = 3. * *p* < 0.05 vs. the Ctr group. (**C**) The changes in mitochondrial ROS in L02 cells were detected by MitoSox staining, N = 3. (**D**) JC-1 staining was used to detect the change in mitochondrial membrane potential in L02 cells, N = 3. (**E**) Serum ALT and AST activities were measured in C57BL/6 mice treated with different doses of APAP, N=10. * *p* < 0.05 vs. the Ctr group, ** *p* < 0.01 vs. the Ctr group. (**F**) HE staining, N = 3. (**G**) Frozen sections of mouse liver tissue were stained with ROS staining solution to measure ROS changes, N = 3.

Excess APAP is mainly metabolized by CYP2E1 enzyme, and we found that CYP2E1 expression was induced in human normal liver cells (L02) and mouse normal liver cells (AML12) following APAP challenges (Figure S1A), indicating that these two cells are suitable for studying APAP toxicity. First, L02 and AML12 cells were treated with different concentrations of APAP for 24 h, and the cell viability decreased with increasing APAP concentration (Figure 1A and Figure S1B). L02 cells were treated with 20 mM APAP, and AML12 cells were treated with 40 mM APAP. Then, indexes related to liver injury were detected, as shown in Figure 1B and Figure S1C. Apoptosis increased with increasing APAP concentration. MitoSox red reagent was used to stain living cells, and gradually enhanced red fluorescence was observed, indicating that mitochondrial ROS increased with increasing APAP concentration (Figure 1C and Figure S1D,F). The fluorescence probe JC-1 was used to detect the mitochondrial membrane potential, and red fluorescence gradually decreased and green fluorescence gradually increased, indicating that the mitochondrial membrane potential decreased with increasing APAP concentration (Figure 1D and Figure S1E,G).

Moreover, as mice are the preferred animals for studying excessive APAP [24,25], we selected C57/BL6 mice as experimental subjects and treated them with 0, 300, 400 and 500 mg/kg APAP for 24 h. Compared with those in the control group, serum ALT and AST levels were increased to varying degrees in all test groups and reached the peak value in the 400 mg/kg APAP group (Figure 1E). HE staining results (Figure 1F) showed that the liver tissue in the control group was normal in shape, with complete hepatic lobule structure, no obvious expansion or extrusion of hepatic sinuses, and round and plump liver cells without deformation or necrosis. No obvious pathological changes were observed in the 300 mg/kg APAP group. In the 400 and 500 mg/kg APAP treatment groups, liver lobules were destroyed, liver cells were denatured, liver cells were necrotic around the central vein, and liver nuclei dissolved and disappeared. Frozen sections were stained with ROS staining solution. Positive expression was indicated by red fluorescence, and the fluorescence intensity was proportional to ROS. The results (Figure 1G) showed that compared with the control group, ROS in the model group increased gradually with increasing APAP dose. The above experimental results indicated that the APAP acute liver injury model was established successfully.

### 3.2. PARK7 Expression Increased in the APAP Acute Liver Injury Model

**Figure 2:**
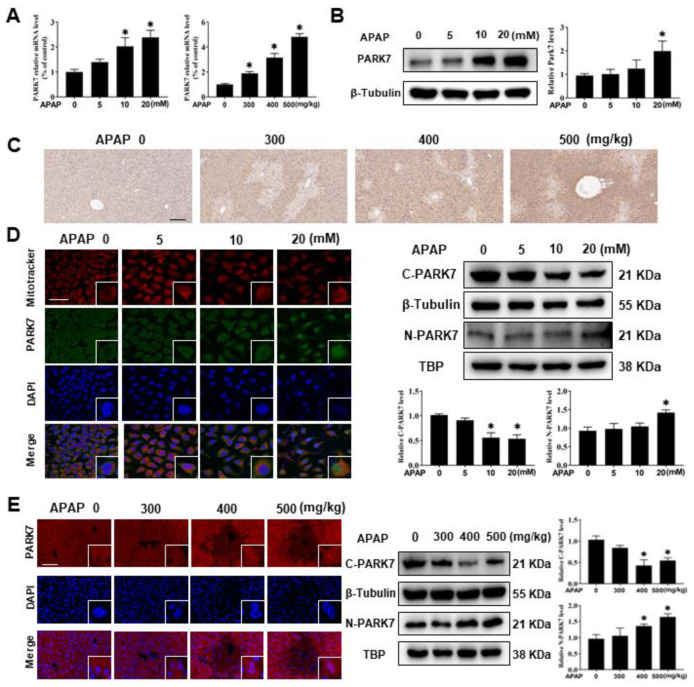
PARK7 expression increased in the APAP acute liver injury model. (**A**) PARK7 mRNA expression was detected in L02 cells and mouse liver tissues, N = 3. * *p* < 0.05 vs. the Ctr group. (**B**) Western blotting (WB) and statistical analysis of PARK7 protein in L02 cells, N = 3. * *p* < 0.05 vs. the Ctr group. (**C**) The expression of PARK7 in liver tissue of mice was detected by immunohistochemical staining, N = 3. (**D**) Immunofluorescence staining and nucleation analysis of L02 cells, N = 3. * *p* < 0.05 vs. the Ctr group. (**E**) Immunofluorescence staining and nucleation analysis of mouse liver tissues, N = 3. * *p* < 0.05 vs. the Ctr group. C represents the cytoplasm and N represents the nucleus.

After induction of the APAP acute liver injury model, we first detected changes in several mitochondrial stress proteins and found that PARK7 changes were most significant (Figure S2A). Then, we detected the expression of PARK7 in L02 cells, AML12 cells and C57 mice, as shown in Figure 2A and Figure S2B. The PCR results showed that PARK7 increased with increasing APAP concentration or dose. Moreover, Western blotting, immunofluorescence and immunohistochemistry of cells or animals also showed that PARK7 increased with increasing APAP concentration or dose (Figure 2B,C and Figure S2C).

Under oxidative stress, PARK7 localized in the cytoplasm translocates to the nucleus [26]. This effect was also observed in the APAP acute liver injury model. As shown in Figure 2D, immunofluorescence and Western blot experiments showed that 20 mM APAP could induce PARK7 to enter the nucleus, and the expression of PARK7 nuclear protein was most significant at this concentration. Immunofluorescence and Western blot analysis of nuclear protein in C57 mouse liver tissue (Figure 2E) also showed that PARK7 nucleation was observed, and the changes were most obvious in the 400 and 500 mg/kg APAP treatment groups.

### 3.3. Silencing PARK7 Alleviated APAP-Induced Acute Liver Injury

Since PARK7 expression increased after APAP treatment, we investigated the function of the PARK7 molecule. Lentivirus was used to silence PARK7 in L02 cells (Figure S3A,B), siRNA was used to silence the PARK7 gene in AML12 cells (Figure S3C–E), and C57 mice were treated with AAV virus (Figure S3F,G). The results showed that the PARK7 silencing/knockdown model was successfully constructed.

**Figure 3:**
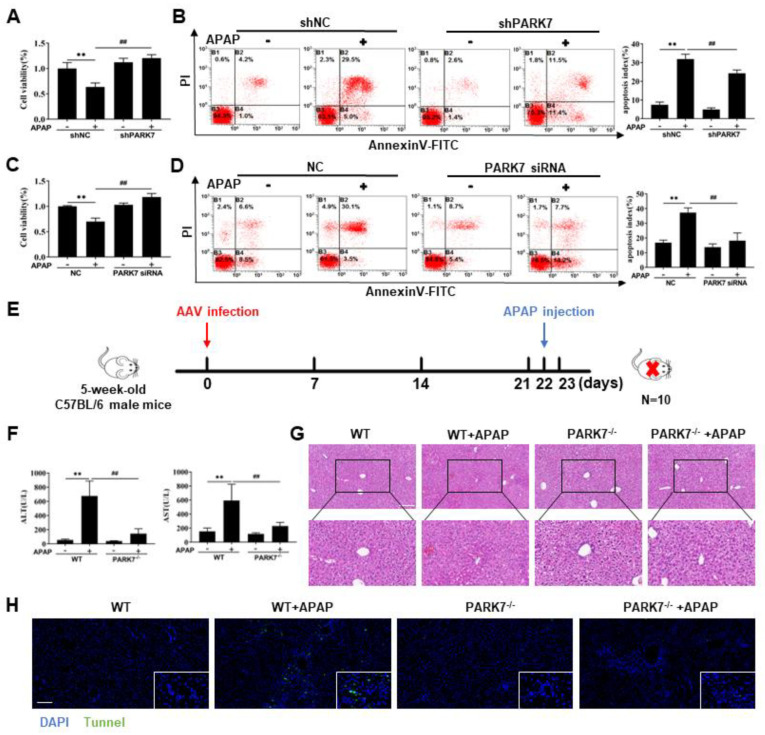
Silencing PARK7 protected against APAP-induced liver injury. (**A**) L02 cell viability under APAP intervention with or without PARK7 silencing, N = 6. (**B**) Apoptosis of L02 cells treated with APAP with or without PARK7 silencing, N = 3. ** *p* < 0.01 vs. shNC group, ## *p* < 0.01 vs. shNC+APAP group. (**C**) Changes in the viability of AML12 cells with or without PARK7 silencing, N = 6. (**D**) Apoptosis of AML12 cells with or without PARK7 silencing, N = 3. ** *p* < 0.01 vs. NC group, ## *p* < 0.01 vs. NC+APAP group. (**E**) Schematic representation of drug intervention in mice. (**F**) Changes in serum ALT and AST in mice after PARK7 knockdown, N = 10. ** *p* < 0.01 vs. WT group, ## *p* < 0.01 vs. WT+APAP group. (**G**) Changes in HE staining after PARK7 knockdown, N = 3. (**H**) TUNEL staining changes after PARK7 knockdown, N = 3.

We then examined functional measures. As shown in Figure 3A,C, compared with that in the shNC group, the cell viability of the shNC+APAP group was decreased. Compared with the shNC+APAP group, the shPARK7+APAP group showed increased cell viability. The results of cell apoptosis analysis (Figure 3B,D) showed that compared with that in the shNC group, the degree of cell apoptosis in the shNC+APAP group was increased. Compared with that in the ShNC+APAP group, the apoptosis rate of shPARK7+APAP cells was decreased. After drug intervention in mice (Figure 3E), compared with those in the WT+APAP group, the serum ALT and AST indexes were significantly reduced in the PARK7 knockdown group, as shown in Figure 3F. The HE staining (Figure 3G) showed that compared with that in the WT+APAP group, the degree of liver injury in mice was reduced in the PARK7 knockdown group. The TUNEL staining results (Figure 3H) showed that compared with that in the WT+APAP group, the degree of liver tissue apoptosis was reduced in the PARK7 knockdown group. These results indicated that silencing PARK7 could attenuate APAP-induced acute liver injury.

### 3.4. Silencing PARK7 Promoted Mitochondrial Autophagy

**Figure 4:**
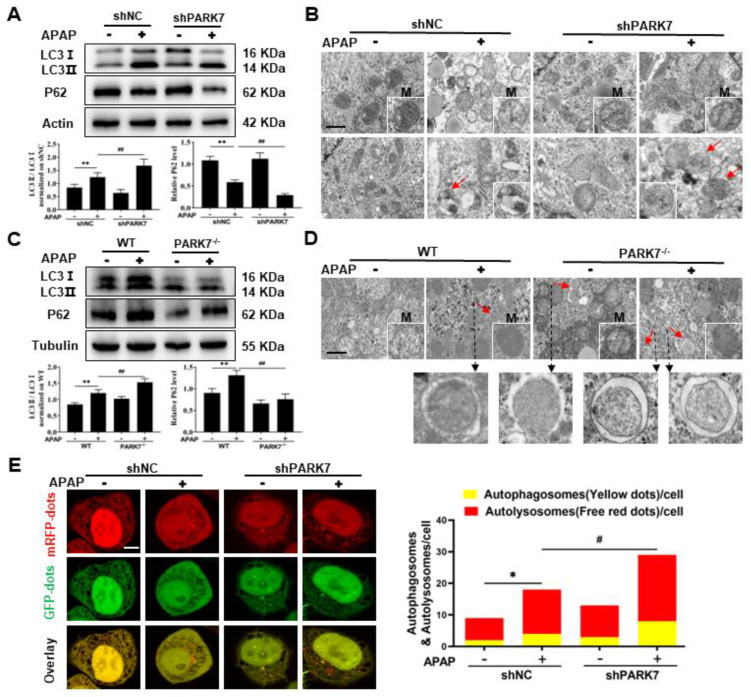
Silencing PARK7 promoted mitochondrial autophagy. (**A**) Changes in LC3 and P62 proteins in APAP-treated L02 cells with and without PARK7 silencing and statistical analysis, N = 3. ** *p* < 0.01 vs. shNC group, ## *p* < 0.01 vs. shNC+APAP group. (**B**) Ultrastructural changes in mitochondria and autophagosomes in L02 cells under APAP intervention with or without PARK7 silencing, N = 3. M represents mitochondria, and the red arrow represents autophagosomes. (**C**) WB and statistical analysis of LC3 and P62 proteins in liver tissue of mice with and without APAP intervention with PARK7 knockdown, N = 3. ** *p* < 0.01 vs. WT group, ## *p* < 0.01 vs. WT+APAP group. (**D**) Ultrastructural changes in mitochondria and autophagosomes in liver tissue of mice with and without APAP intervention with PARK7 knockdown, N = 3. Black arrows indicate enlarged images of autophagosomes. (**E**) Changes in autophagy flux in APAP-treated L02 cells with or without PARK7 silencing, N = 3. * *p* < 0.05 vs. shNC group, # *p* < 0.05 vs. shNC+APAP group.

To investigate the relationship between PARK7 and mitochondrial autophagy in APAP-induced acute liver injury, we examined the changes in key autophagy proteins, mitochondrial ultrastructure, and autophagosomes. As shown in Figure 4A, compared with that in the shNC group, the expression of LC3 protein in the shNC+APAP group increased, while the expression of P62 protein decreased. Compared with that in the shNC+APAP group, LC3 protein expression was increased and P62 protein expression was decreased in the shPARK7+APAP group. Electron microscopy (Figure 4B) showed that mitochondria in the shNC group were oval or short rod-shaped with uniform size, and no autophagosomes were produced. In the shNC+APAP group, mitochondria swelled, matrix density decreased, and autophagosomes were activated. The shPARK7 group had elliptical mitochondria without autophagosomes. The shPARK7+APAP group exhibited reduced mitochondrial swelling and increased autophagosome formation.

The WB results for mouse liver tissue are shown in Figure 4C. Compared with the WT group, LC3 and P62 protein expression increased in in the WT+APAP group. Compared with the WT+APAP group, LC3 protein expression in the PARK7^−/−^+APAP group increased, and P62 protein expression decreased. P62 protein expression may have increased because P62 is a stress protein, whose expression is greatly up-regulated under toxic stimulation or stress conditions; this process is mainly regulated by transcription factor EB (TFEB). Electron microscopy of mouse liver tissue (Figure 4D) showed that autophagosomes were also present in the PARK7 knockdown group; the other changes were basically consistent with those observed in L02 cells by electron microscopy.

To further observe the phenomenon of autophagy, we used the autophagy double-label adenovirus to monitor the changes in autophagic flux in real time (Figure 4E) and observed the strength of autophagic flux by spot counting. Compared with the shNC group, autophagic flux was activated in the shNC+APAP group. Compared with the shNC+APAP group, autophagic flux was enhanced in the shPARK7+APAP group. After treatment with chloroquine (10 μM), the expression of LC3 in both the shNC+APAP group and shPARK7+APAP group increased (Figure S4A,B), indicating that chloroquine inhibits the fusion of autophagosomes and lysosomes, leading to the failure of autophagy. Therefore, PARK7 inhibits mitochondrial autophagy in APAP-induced acute liver injury models, but silencing PARK7 can reverse this phenomenon.

### 3.5. The Protective Effect of PARK7 Silencing Was Not Due to Antioxidant Activity

**Figure 5:**
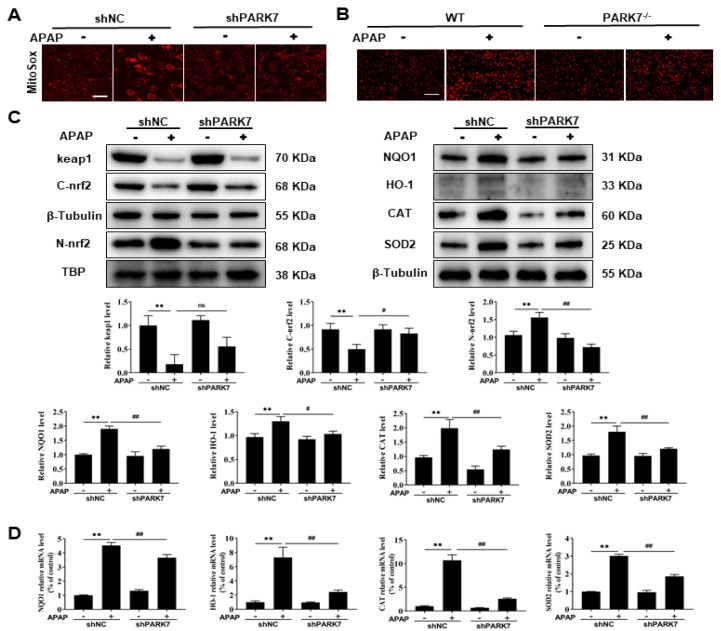
The protective effect of PARK7 silencing was not due to antioxidant activity. (**A**) Changes in mitochondrial ROS in L02 cells treated with APAP with or without PARK7 silencing, N = 3. (**B**) Changes in mitochondrial ROS in liver tissue of mice with or without APAP intervention with PARK7 knockdown, N = 3. (**C**) WB and statistical analysis of Keap1, Nrf2, NQO1, HO-1, SOD2 and CAT proteins under APAP intervention with or without PARK7 silencing, N = 3. (**D**) Changes in mRNA levels of NQO1, HO-1, SOD2 and CAT under APAP intervention with or without PARK7 silencing, N = 3. ** *p* < 0.01 vs. ShNC group, # *p* < 0.05 vs. ShNC+APAP group, ## *p* < 0.01 vs. ShNC+APAP group. C represents the cytoplasm and N represents the nucleus.

Subsequently, we wondered whether the protective effect of PARK7 silencing was caused by the enhancement of the antioxidant effect. We first detected mitochondrial ROS, and the results (Figure 5A and Figure S5A,B) showed that mitochondrial ROS increased in the shNC+APAP group compared with the shNC group. Compared with that in the shNC+APAP group, mitochondrial ROS decreased in the shPARK7+APAP group. The ROS changes in frozen sections of mouse liver tissue (Figure 5B) were consistent with the cell results. As shown in Figure 5C, the antioxidant protein Nrf2 was nucleated under APAP stimulation and dissociated from Keap1. Compared with that in the shNC group, the expression of Keap1 was decreased and the expression of Nrf2 was increased in the shNC+APAP group. Compared with that in the shNC+APAP group, the expression of Keap1 was increased and the expression of NRF2 was decreased in the shPARK7+APAP group. The expression levels of NQO1, HO-1, SOD2 and CAT in the shNC+APAP group were increased compared with those in the shNC group (Figure 5C,D). Compared with those in the shNC+APAP group, the expression levels of NQO1, HO-1, SOD2 and CAT were decreased in the shPARK7+APAP group. Therefore, the protective effect of PARK7 silencing was not caused by increased antioxidant activity.

### 3.6. The Protective Effect of Silencing PARK7 Was Related to Mitochondrial Synthesis and Metabolic Reprogramming

**Figure 6:**
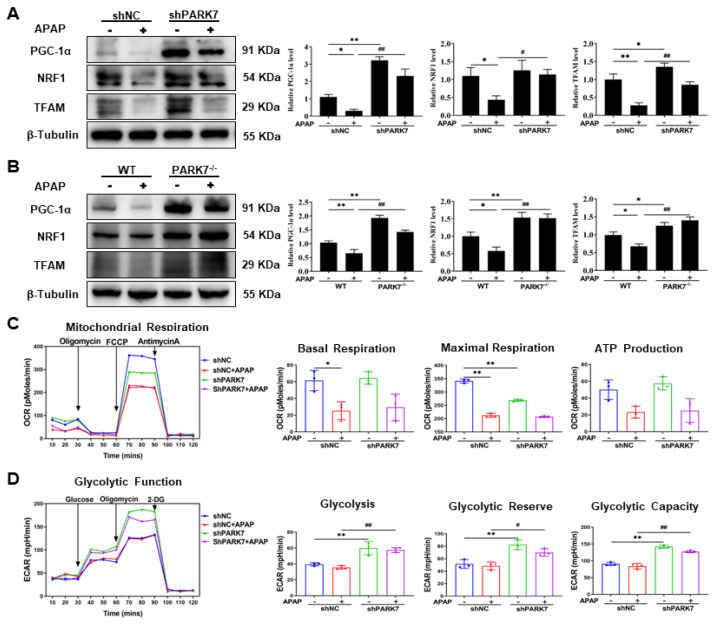
Changes in mitochondrial-related indexes after PARK7 silencing. (**A**) Changes in mitochondrial synthesis proteins in APAP-treated L02 cells with and without PARK7 silencing and statistical analysis, N = 3. * *p* < 0.05 vs. ShNC group, ** *p* < 0.01 vs. ShNC group, # *p* < 0.05vs. ShNC+APAP group, ## *p* < 0.01 vs. ShNC+APAP group. (**B**) WB and statistical analysis of the mitochondrial synthetic proteins PGC-1α, NRF1 and TFAM in the liver tissue of mice with or without APAP intervention with PARK7 knockdown, N = 3, * *p* < 0.05 vs. WT group, ** *p* < 0.01 vs. WT group, ## *p* < 0.01 vs. WT+APAP group. (**C**,**D**) Changes in mitochondrial respiratory function and glycolytic function under APAP intervention with or without PARK7 silencing, N = 3. * *p* < 0.05 vs. shNC group, ** *p* < 0.01 vs. shNC group, # *p* < 0.05 vs. shNC+APAP group, ## *p* < 0.01 vs. shNC+APAP group.

Peroxisome proliferator-activated receptor γ coactivator 1α (PGC-1α) plays a key role in regulating mitochondrial biogenesis and oxidative metabolism [27]. The protein expression levels of PGC1-α and its downstream nuclear respiratory factor 1 (NRF1) and mitochondrial transcription factor A (TFAM) were detected, as shown in Figure 6A. Compared with the shNC group, the expression levels of PGC1-α, NRF1 and TFAM were all decreased in the shNC+APAP group. Compared with the shNC+APAP group, the expression levels of PGC1-α, NRF1 and TFAM were increased in the shPARK7+APAP group. The gene expression levels of PGC1-α, NRF1 and TFAM were basically consistent with the protein expression levels (Figure S6A). The protein expression levels of PGC-1α, NRF1 and TFAM in mouse liver tissues (Figure 6B) were consistent with the cellular results.

The Seahorse experiment results showed (Figure 6C,D) that basal respiration, maximal respiration and ATP production decreased significantly in the shNC+APAP group compared with the shNC group. Silencing PARK7 did not reverse this phenomenon. Compared with those in the shNC group and shNC+APAP group, the glycolysis, glycolysis capacity and glycolytic reserve of PARK7-silenced cells were significantly increased. As shown in Figure S6B, compared with that in the shNC group, the ECAR increased after PARK7 silencing, indicating that the main energy metabolism pathways of the cells shifted from oxidative phosphorylation to glycolysis.

## 4. Discussion

Mitochondria, as essential organelles in cells, are the main sites of ROS production. In recent years, oxidative stress and mitochondrial dysfunction have been considered key events in the pathogenesis of hepatotoxicity of APAP. Moreover, it is generally believed that there is a causal relationship between mitochondrial dysfunction and oxidative stress caused by excessive APAP [6]. In addition, mitochondrial quality control systems, including mitochondrial autophagy and mitochondrial synthesis, have attracted increasing attention [10]. The results of this study mainly demonstrated that silencing PARK7 initiated mitochondrial autophagy to remove damaged mitochondria and reduced ROS level. In addition, silencing PARK7 promoted mitochondrial synthesis and replaced damaged mitochondria.

Repeated doses of acetaminophen given for therapeutic reasons have been reported to cause hepatotoxicity in adults and children. APAP blood levels in these cases ranged from 0 to 23 mg/mL [28]. A concentration of 23 mg/mL is equivalent to 152 mM, so the concentration of 20 or 40 mM APAP given to the cells in this study should be achievable for the patient. Toxic doses of APAP can lead to mitochondrial damage [29]. We observed an increase in mitochondrial ROS levels and a decrease in mitochondrial membrane potential in cells treated with gradient concentrations of APAP. In a mouse model of APAP liver injury, serum ALT and AST activities increased after 24 h of APAP treatment at a toxic dose. Liver histopathology showed significant changes in central lobular necrosis and increased ROS levels in frozen sections. Lobular central hepatocytes have a strong hepatotoxicity susceptibility to APAP, which may be due to the enhanced expression of CYP2E1 and the low storage level of GSH [30].

Under APAP stimulation, mitochondrial oxidative stress was enhanced, and PARK7 expression increased with increasing APAP concentration. Recent studies have demonstrated reduced severity of liver injury and reduced mortality rates in PARK7 knockout mice [31]. Similarly, we observed this phenomenon in cell and animal models.

Studies have shown that autophagy plays a protective role in APAP-induced liver injury, and autophagy is enhanced after excessive APAP treatment and acts as a defense mechanism to remove damaged mitochondria [32,33,34]. Mitochondria are organelles that can regulate autophagy and are the main source of ROS in the autophagy signaling pathway. Mitochondrial autophagy protects cells from oxidative damage by eliminating the source of oxidative stress [35]. Mitochondrial autophagy can protect against APAP-induced acute liver injury in mice [36]. Our results showed that compared with that in the control group, the LC3 level increased, the P62 level decreased and the number of autophagosomes increased after APAP treatment. As a marker of autophagy, LC3 participates in the formation of autophagosomes and is used to monitor autophagy activity [37]. The LC3-II/I ratio is used to estimate the level of autophagy. P62 is a selective autophagy adapter protein that also degrades with the substrate during lysosomal degradation. Therefore, an increased level of P62 is generally considered a marker of inhibition of autophagy activity [38]. Furthermore, we found that autophagy was significantly enhanced after silencing PARK7 compared with APAP treatment alone. On the one hand, LC3 expression increased, and the electron microscopy results showed that the accumulation of autophagosomes increased. On the other hand, autophagic flux was enhanced after infection with mRFP-GFP-LC3. In summary, our results suggest that mitochondrial autophagy is enhanced after silencing PARK7. In addition, ROS can regulate autophagy by directly affecting the core mechanism of autophagy [39]. Therefore, autophagy activation may be a protective response to oxidative stress after APAP exposure.

Oxidative stress refers to a state of imbalance between oxidation and antioxidant effects in the body. APAP leads to mitochondrial stress and produces a large number of ROS. Nrf2 is known to be a core regulator of antioxidant activity and interacts with Keap1. Under oxidative stress, stimulated by mitochondrial ROS, Nrf2 is separated from Keap1 and enters the nucleus to bind with antioxidant response elements (AREs), triggering the expression of downstream antioxidant enzymes [40]. High doses of APAP metabolites not only deplete glutathione reserves but also cause the breakdown of the liver’s antioxidant defense system [41]. We investigated the expression of Nrf2 and its downstream antioxidant enzymes, and the results showed that compared with those in the normal control group, the expression levels of Nrf2 and its downstream factors NQO1, HO-1, SOD2 and CAT were increased after APAP treatment. However, after silencing PARK7, the expression levels of Nrf2 and its downstream antioxidant molecules were decreased by APAP intervention. These results indicated that when a large number of mitochondrial ROS were produced by APAP stimulation, the antioxidant capacity was enhanced to initiate the protective effect; when PARK7 was silenced, mtROS production was reduced, and the antioxidant effect was weakened. In conclusion, the protective effect of silencing PARK7 was not mediated by antioxidants.

The mitochondrial quality control system is crucial for maintaining mitochondrial function [42]. As mitochondria are the main targets of NAPQI, mitochondrial quality control, which includes mitochondrial biogenesis and mitochondrial autophagy, has become an important strategy to reduce ROS and liver injury [43]. Mitochondrial biogenesis is crucial for the normal regeneration and homeostasis of cells, and it maintains key cellular processes such as respiration and metabolism by promoting mitochondrial regeneration [44]. Induction of mitochondrial biogenesis protects against APAP-induced liver injury [45]. Peroxisome proliferator-activated receptor γ-coactivator 1α (PGC-1α) is an important regulator of mitochondrial biogenesis [27] that controls a series of downstream targets, including NRF1 and TFAM. With excessive APAP, PGC-1 α expression is decreased [46]. Our results showed that the expression of PGC1-α, NRF1 and TFAM decreased after APAP intervention compared with the control group. However, the expression of PGC1-α, NRF1 and TFAM increased after PARK7 silencing and APAP intervention. These results indicated that APAP, through NAPQI, consumed GSH, caused oxidative stress, disrupted the structure and function of mitochondria and blocked the generation of mitochondria, while silencing PARK7 promoted the generation of mitochondria. In addition, the Seahorse assay showed that the energy metabolism pathway in cells after PARK7 silencing shifted from oxidative phosphorylation to glycolysis. These results indicate that in a hypoxic environment, PARK7-silenced cells change their glucose metabolism mode from oxidative phosphorylation to glycolysis, which enables cells to survive in a hypoxic environment and maintain the balance of redox reactions; this is referred to as the metabolic reprogramming phenomenon. Switching cells from aerobic metabolism to anaerobic glycolysis is a protective mechanism that reduces mitochondrial ROS production.

## 5. Conclusions

**Figure 7:**
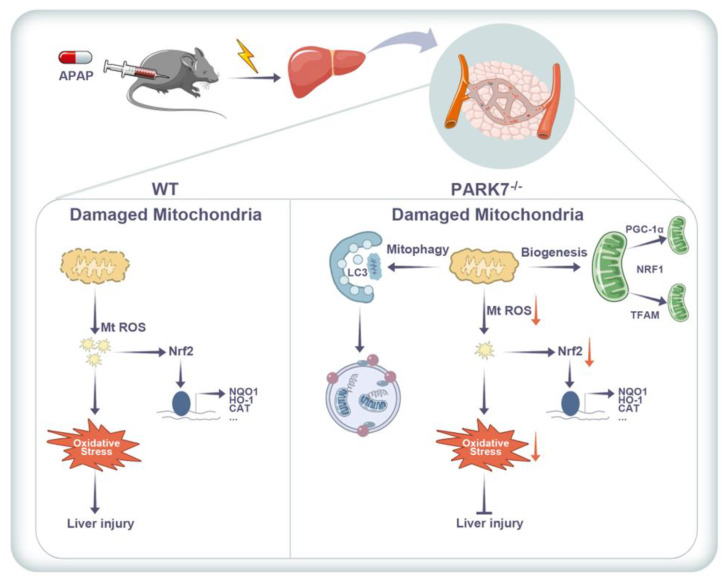
Scheme of this study.

In conclusion, this study suggests that silencing PARK7 enhances mitochondrial quality control. On the one hand, it removes damaged mitochondria by enhancing autophagy. On the other hand, by increasing mitochondrial biosynthesis to renew mitochondria, the two work together to protect APAP-induced acute liver injury (Figure 7). Therefore, we propose that PARK7 may be a potential therapeutic target in APAP-induced acute liver injury.

After silencing PARK7, the level of ROS decreased by activating mitophagy to clear damaged mitochondria. In addition, silencing PARK7 promoted mitochondrial synthesis and replaced damaged mitochondria.

## Data Availability

All of the data is contained within the article and the Supplementary Materials.

## Supplementary Materials

The following supporting information can be downloaded at: https://www.mdpi.com/article/10.3390/antiox11112128/s1, Figure S1: Establishment of the APAP acute liver injury model. (A) The mRNA expression of CYP2E1 was detected in C57 mice, L02 cells and AML12 cells, and the groups were control and APAP groups, N = 3. * *p* < 0.05 vs. the Ctr group. ** *p* < 0.01 vs. the Ctr group. (B) AML12 cells were treated with gradient concentrations of APAP to detect changes in cell viability, N = 6. * *p* < 0.05 vs. the Ctr group. (C) AML12 cell apoptosis was detected by AnnexinV/PI double staining, N = 3. * *p* < 0.05 vs. the Ctr group. (D) MitoSox staining was used to detect the change of mitochondrial ROS in AML12 cells, N = 3. (E) Changes in mitochondrial membrane potential of AML12 cells detected by JC-1 staining, N = 3. (F) MitoSox stained cells tested by flow cytometry. (G) JC-1 stained cells tested by flow cytometry; Figure S2: PARK7 expression increased in the APAP acute liver injury model. (A) mRNA changes of mitochondrial stress molecules in L02 cells, N = 3. * *p* < 0.05 vs. the Ctr group. (B) Expression of PARK7 mRNA in AML12 cells, N = 3. * *p* < 0.05 vs. the Ctr group; (C) Immunofluorescence staining of PARK7 in AML12 cells, N = 3; Figure S3: Validation of PARK7 silencing efficiency. (A) PARK7 silencing was verified by WB and PCR in L02 cells, N = 3. * *p* < 0.05 vs. the Ctr group. (B) Changes in PARK7 expression detected by WB and PCR in response to APAP intervention in groups with or without PARK7 silencing, N = 3. ** *p* < 0.01 vs. shNC group, ## *p* < 0.01 vs. shNC+APAP group. (C) PARK7 silencing was verified by WB and PCR in AML12 cells, N = 3. * *p* < 0.05 vs. the Ctr group. (D) PCR changes in PARK7 in AML12 cells in response to APAP intervention with or without PARK7 silencing, N = 3. ** *p* < 0.01 vs. NC group, ## *p* < 0.01 vs. NC+APAP group. (E) The results of immunofluorescence staining of PARK7 in AML12 cells under the intervention of APAP with or without PARK7 silencing, N = 3. (F) C57BL/6 mice were transfected with AAV virus, and liver tissue sections were used to detect the transfection effect with fluorescence. (G) PCR and WB validation of the PARK7 knockdown effect in C57 mice, N = 3. ** *p* < 0.01 vs. WT group; Figure S4: Silencing PARK7 promoted mitochondrial autophagy. (A) Changes in cell viability were detected after L02 cells were treated with different concentrations of chloroquine, N = 6. (B) LC3 protein changes in cells with and without PARK7 silencing and APAP and chloroquine intervention, N = 3; Figure S5: Changes in antioxidant-related proteins after silencing PARK7. (A) Changes of mitochondrial ROS in AML12 cells interfered with APAP with or without PARK7 silencing, N = 3. (B) MitoSox stained cells tested by flow cytometry; Figure S6: Changes in mitochondrial-related indexes after PARK7 silencing. (A)mRNA changes of mitochondrial syn-thetic genes PGC-1α, NRF1 and TFAM under APAP intervention with or without PARK7 silencing, N = 3. ** *p* < 0.01 vs. ShNC group, # *p* < 0.05 vs. ShNC+APAP group, ## *p* < 0.01 vs. ShNC+APAP group. (B) Changes in mitochondrial ECAR and OCR under APAP intervention with or without PARK7 silencing.
