# Supplementary material for: Targeting PARK7 Improves Acetaminophen-Induced Acute Liver Injury by Orchestrating Mitochondrial Quality Control and Metabolic Reprogramming
Source: Antioxidants (Basel). 2022 Oct 28;11(11):2128. doi: 10.3390/antiox11112128 (PMC9686963; doi:10.3390/antiox11112128)
Supplement: Supplementary file 1 [file antioxidants-11-02128-s001.zip › antioxidants-1891626--supplementary/antioxidants-1891626--supplementary.pdf]

Supplementary Materials

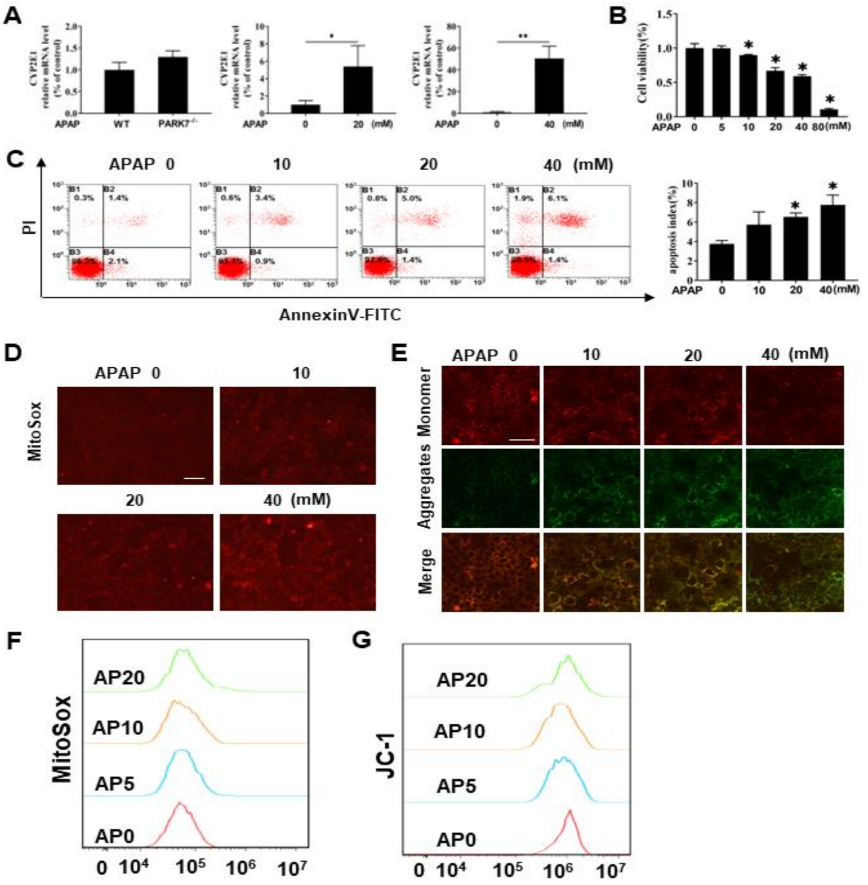

Figure S1. Establishment of the APAP acute liver injury model. (A) The mRNA expression of CYP2E1 was detected in C57 mice, L02 cells and AML12 cells, and the groups were control and APAP groups, N=3. *P<0.05 vs. the Ctr group. **P<0.01 vs. the Ctr group. (B) AML12 cells were treated with gradient concentrations of APAP to detect changes in cell viability, N=6. *P<0.05 vs. the Ctr group. (C) AML12 cell apoptosis was detected by AnnexinV/PI double staining, N=3. *P<0.05 vs. the Ctr group. (D) MitoSox staining was used to detect the change of mitochondrial ROS in AML12 cells, N=3. (E) Changes in mitochondrial membrane potential of AML12 cells detected by JC-1 staining, N=3. (F) MitoSox stained cells tested by flow cytometry. (G) JC-1 stained cells tested by flow cytometry.

The primer information used in Figure. s1A is as follows:
Actin(human)-F: CTCCATCCTGGCCTCGCTGT
Actin(human)-R: GCTGTCACCTTCACCGTTCC
Actin(Mus)-F: AACAGTCCGCCTAGAAGCAC
Actin(Mus)-R: CGTTGACATCCGTAAAGACC
CYP2E1(human)-F: TCGAAGAGAAGCTCCATGAAGAA
CYP2E1(human)-R: GTGATGAACCGCTGAATCTCATG
CYP2E1(Mus)-F: CTGAGATATGGGCTCCTGATTCT
CYP2E1(Mus)-R: GAAGGGACGAGGTTGATGAATCT

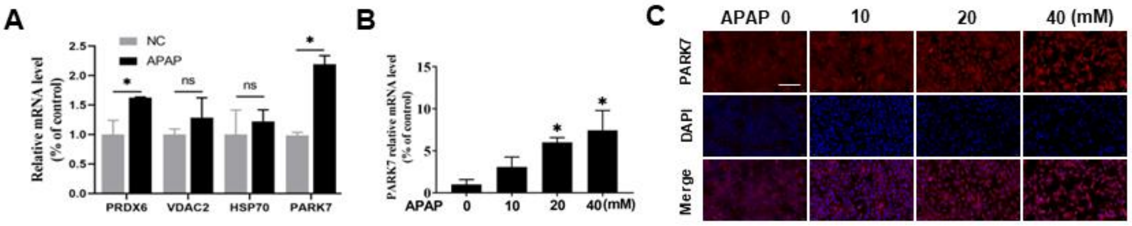

Figure S2. PARK7 expression increased in the APAP acute liver injury model. (A) mRNA changes of mitochondrial stress molecules in L02 cells, N=3. *P<0.05 vs. the Ctr group. (B) Expression of PARK7 mRNA in AML12 cells, N=3. *P<0.05 vs. the Ctr group; (C) Immunofluorescence staining of PARK7 in AML12 cells, N=3.

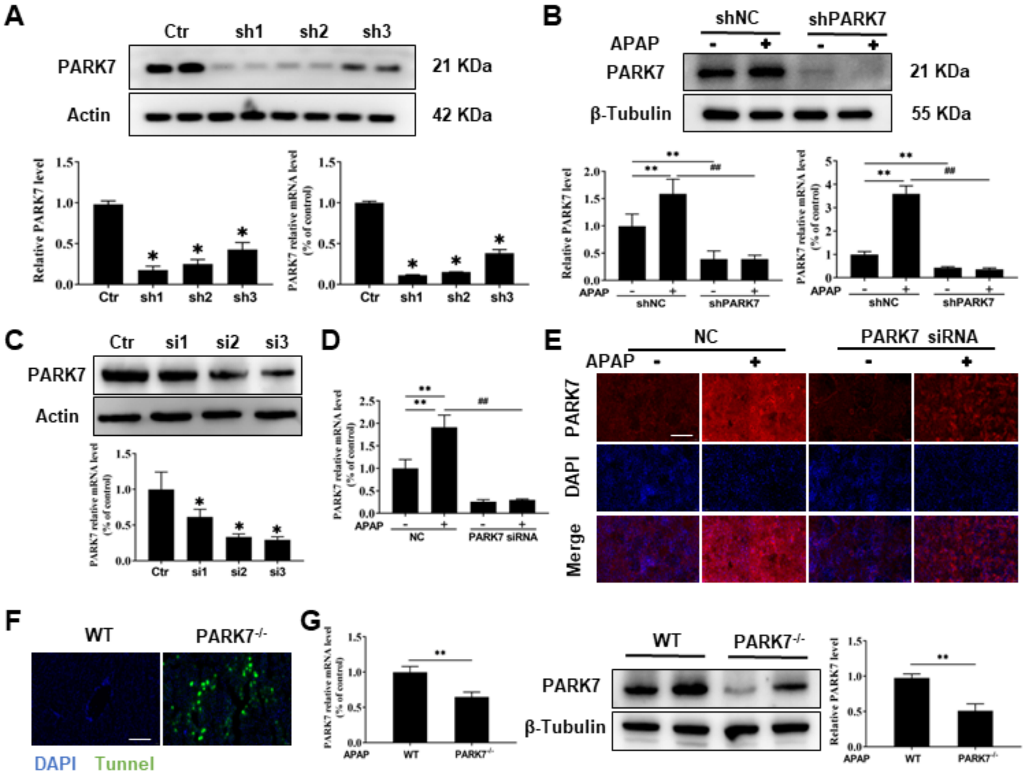

Figure S3. Validation of PARK7 silencing efficiency. (A) PARK7 silencing was verified by WB and PCR in L02 cells, N=3. *P < 0.05 vs. the Ctr group. (B) Changes in PARK7 expression detected by WB and PCR in response to APAP intervention in groups with or without PARK7 silencing, N=3. **P < 0.01 vs. shNC group, ## P < 0.01 vs. shNC+APAP group. (C) PARK7 silencing was verified by WB and PCR in AML12 cells, N=3. *P < 0.05 vs. the Ctr group. (D) PCR changes in PARK7 in AML12 cells in response to APAP intervention with or without PARK7 silencing, N=3. **P < 0.01 vs. NC group, ## P < 0.01 vs. NC+APAP group. (E) The results of immunofluorescence staining of PARK7 in AML12 cells under the intervention of APAP with or without PARK7 silencing, N=3. (F) C57BL/6 mice were transfected with AAV virus, and liver tissue sections were used to detect the transfection effect with fluorescence. (G) PCR and WB validation of the PARK7 knockdown effect in C57 mice, N=3. **P < 0.01 vs. WT group.

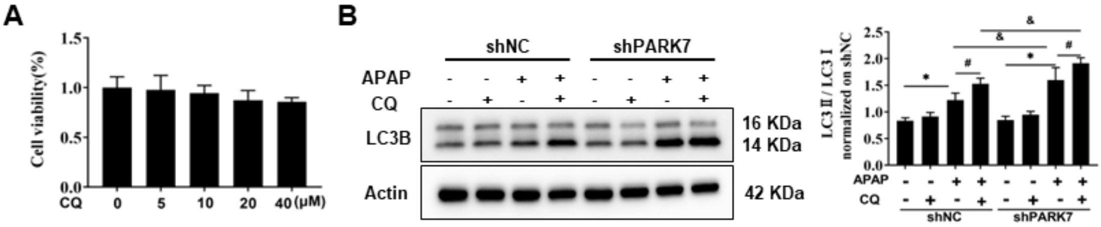

Figure S4. Silencing PARK7 promoted mitochondrial autophagy. (A) Changes in cell viability were detected after L02 cells were treated with different concentrations of chloroquine, N=6. (B) LC3 protein changes in cells with and without PARK7 silencing and APAP and chloroquine intervention, N=3.

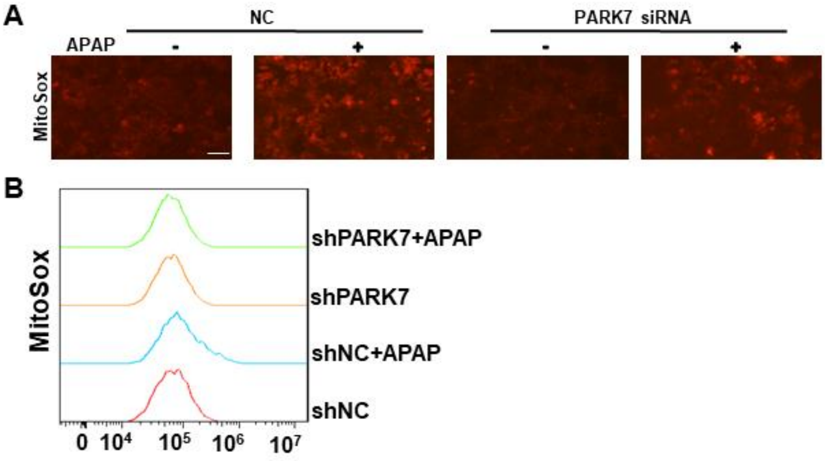

Figure S5. Changes in antioxidant-related proteins after silencing PARK7. (A) Changes of mitochondrial ROS in AML12 cells interfered with APAP with or without PARK7 silencing, N=3. (B) MitoSox stained cells tested by flow cytometry.

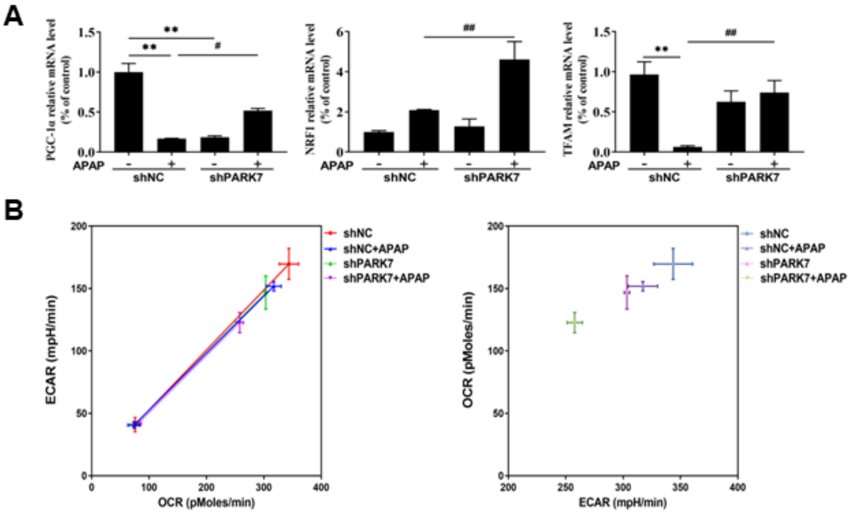

Figure S6. Changes in mitochondrial-related indexes after PARK7 silencing. (A)mRNA changes of mitochondrial synthetic genes PGC-1α, NRF1 and TFAM under APAP intervention with or without PARK7 silencing, N=3. $^{**}P < 0.01$ vs. ShNC group, # $P < 0.05$ vs. ShNC+APAP group, ## $P < 0.01$ vs. ShNC+APAP group. (B) Changes in mitochondrial ECAR and OCR under APAP intervention with or without PARK7 silencing.
